# Genome-Wide Identification, Characterization, and Expression Analysis of the U-Box Gene Family in Cucumber (*Cucumis sativus*)

**DOI:** 10.3390/plants14121801

**Published:** 2025-06-12

**Authors:** Quanqing Chen, Tian Zhao, Hao Song, Siyuan Sha, Jun Ma, Ruihan Zhang, Weiwen Kong, Shuying Yang, Jinglan Liu, Yiping Wang

**Affiliations:** 1 College of Plant Protection, Yangzhou University, Yangzhou 225009, China; 2 Joint International Research Laboratory of Agriculture and Agri-Product Safety of the Ministry of Education, Yangzhou University, Yangzhou 225009, China

**Keywords:** *Cucumis sativus*, plant U-box, E3 ligase, ARM repeats, biotic stress, gene expression, subcellular localization

## Abstract

Plant U-box (PUB) E3 ubiquitin ligases have undergone significant expansion compared to their fungal and animal counterparts. These E3 ligases play critical roles in diverse biological processes, including responses to biotic and abiotic stresses. However, systematic identification of *PUB* genes in cucumber (*Cucumis sativus* L.) has been lacking, and their expression and functional characterization remain largely unexplored. Leveraging the recently released near-complete cucumber genome, we identified 53 putative PUB proteins classified into eight distinct groups based on domain architecture. The molecular weights of CsPUBs range from 26 to 166 kilodaltons (kDa). Exon numbers in *CsPUB* genes vary substantially, with *CsPUB48* containing a maximum of 17 exons, while 18 *CsPUB* genes harbor only a single exon. Chromosomal distribution of *CsPUBs* is uneven, with Chr 3 harboring the highest density (12 genes) and Chr 7 the lowest (1 gene). Notably, tandem duplications (e.g., *CsPUB29*-*CsPUB36* and *CsPUB18*-*CsPUB49*) and seven collinear gene pairs were identified, suggesting evolutionary diversification. Promoter regions of *CsPUBs* are enriched with *cis*-regulatory elements linked to plant growth and development, phytohormone, stress responses, light, and so on, implying their regulatory roles in various biological processes. Expression profiling revealed tissue-specific patterns and differential regulation of multiple *CsPUBs* under stress conditions. Subcellular localization studies demonstrated that CsPUBs target diverse organelles, with some localizing to punctate structures potentially representing uncharacterized compartments. Collectively, this systematic analysis establishes a comprehensive framework for understanding particular CsPUB functions.

## 1. Introduction

The ubiquitin/26S proteasome system (UPS), which mediates the degradation of ubiquitinated target proteins, serves crucial regulatory functions in diverse biological processes, including growth, development, and stress responses [1]. Ubiquitination of a target protein is mainly executed by three enzymes, including E1 ubiquitin (UB) activating enzyme, E2 UB binding enzyme, and E3 UB ligase. An E1 activates UB in the presence of ATP supplies and then transfers it to an E2. Then, Ub is attached to the substrate protein by an E3 ligase, which determines the substrate specificity in the ubiquitination process [2]. Ub-conjugated substrate is then transferred to the 26S proteasome, which finally degrades the protein [3].

The ubiquitin-proteasome system (UPS) plays a crucial role in cellular activities, and its related genes constitute a significant portion of the total genes in eukaryotic genomes. For instance, in *Arabidopsis thaliana*, approximately 6% of the genome, which corresponds to about 1600 genes, encodes key components of the UPS [1]. These components include two E1 enzymes, 37 E2 enzymes, and more than 1400 E3 ligases [1,4,5]. Based on their molecular composition and activation mechanisms, E3 ubiquitin ligases are primarily grouped into two main categories: multi-subunit forms and single-subunit forms. The latter is further divided into three families: homologous to E6-associated protein carboxyl terminus (HECT)-type E3 ligases, really interesting new gene (RING) finger-type E3 ligases, and U-box domain-type E3 ligases [6].

U-box proteins feature a distinctive U-box domain, comprising roughly 75 amino acids. This domain is evolutionarily related to the RING finger domain but differs due to the absence of the zinc-binding cysteine and histidine residues that are hallmark features of the RING finger [7]. There are two and eight u-box proteins in yeast and humans [8,9], respectively. In plants, nevertheless, the u-box proteins have undergone a significant expansion in comparison to their fungal and animal counterparts. For instance, the Arabidopsis genome encodes 64 U-box-containing proteins [10], while *Chlamydomonas reinhardtii*, rice (*Oryza sativa* L.), barley (*Hordeum vulgare* L.), tomato (*Solanum lycopersicum* L.), and soybean (*Glycine max* L.) have 30 [11], 77 [12], 67 [13], 62 [14], and 125 [15] members, respectively. The plant U-box protein (PUBs) family has been categorized into 13 distinct groups based on the presence and arrangement of their domains. Most PUB proteins contain an armadillo repeat (ARM) domain that facilitates protein–protein interactions. Other typical domains include ubiquitin fusion degradation 2 (UFD2), the U-box N-terminal domain (UND), serine/threonine kinase domain (Kinase), WD40 repeat (WD40), middle domain of eukaryotic initiation factor 4G (MIF4G), tetratricopeptide repeat (TPR), and peptidylprolyl isomerase (PPIase) [16,17].

Emerging evidence indicates that PUB proteins are crucial components in numerous physiological processes, including growth and development, abiotic stress response, and particularly in plant immunity. ARC1, the *Brassica napus* PUB protein, functions downstream of the *S* receptor kinase (SRK) to regulate self-incompatibility by promoting protein ubiquitination and proteasomal degradation of compatibility factors in the pistil [18]. In Arabidopsis, *AtPUB4* plays a role in the CLAVATA3 (CLV3)/endosperm surrounding region (ESR)-related (CLE) signaling pathway to regulate root cell proliferation and columella stem cell maintenance [19]. Additionally, it interacts with extra-large G-proteins (XLGs) to modulate cytokinin responses, stamen development, and male fertility [20]. Furthermore, *AtPUB4* has been shown to associate with several key components of pattern-triggered immunity (PTI) to confer disease resistance in both Arabidopsis and tomato [21,22]. Besides *AtPUB4* and its closely related *AtPUB2*, a couple of *PUBs* have been confirmed to play positive or negative roles in plant immunity in Arabidopsis. *AtPUB12* and *AtPUB13* facilitate the polyubiquitination of FLS2 to promote its degradation upon flagellin induction, thus serving as negative regulators to avoid excessive or prolonged activation of plant immune responses [23]. *AtPUB25* and *AtPUB26* play critical roles in maintaining BIK1 homeostasis by targeting non-activated BIK1 for degradation through ubiquitination [24]. Additionally, they also degrade *AtMYB6*, which is a positive regulator of disease resistance against the hemibiotrophic pathogen *Verticillium dahliae* [25]. In rice, several *PUBs*, including *OsSPL11*, *OsCIE1*/*OsPUB12*, *OsPUB44*, and *OsPUB73*, have been shown to regulate disease resistance against *Xanthomonas oryzae* pv. *oryzae* or *Magnaporthe oryzae* by ubiquitinating their substrates [26,27,28,29,30,31].

Despite the extensive research on *PUBs* in Arabidopsis and rice, there is still a lack of clear identification and functional studies for *PUBs* in many plant species, such as cucumber (*C. sativus*). Recently, a near-complete cucumber reference genome has been released, which provides valuable resources for gene functioning and evolution studies [32]. In this work, we systematically investigated the *PUB* gene family in cucumber by analyzing their annotation, protein categories, gene and protein structures, evolutionary relationships, physicochemical properties, chromosomal localization, collinear analysis, organ–tissue expression profiles, responses to biotic and abiotic stresses, and subcellular localization patterns. These findings provide crucial insights into the functional characterization of PUB E3 ubiquitin ligases in cucumber (*C. sativus*), while also identifying potential gene targets for improving stress resistance through modern molecular breeding approaches, including genome-editing technologies.

## 2. Results

### 2.1. Genome-Wide Identification of U-Box Gene Family Members in Cucumber

**Figure 1:**
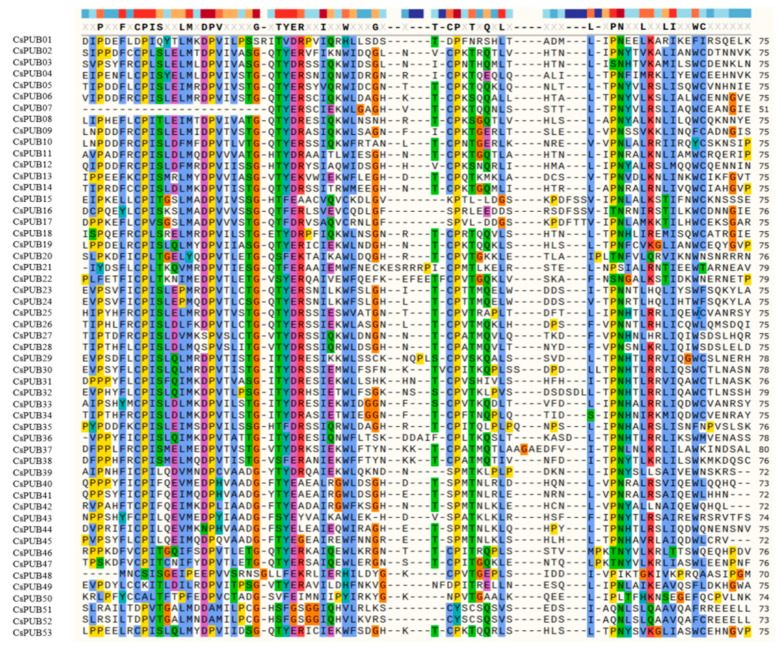
Comparative analysis of U-box sequences in CsPUBs. A comparative analysis of the U-box domain of CsPUB proteins with color-coding highlighting their attributes and conservation. Color legend: The conservation gradient (top) ranges from warm tones (high conservation) to cool tones (low conservation). Amino acid properties are indicated by: blue—hydrophobic, green—polar, red—basic, purple—acidic, teal—aromatic; residues with undefined properties retain default coloring.

In this study, we systematically characterized the PUB protein family in *C. sativus* through integrated genomic analyses. We identified 53 *CsPUB* genes in the cucumber genome through comprehensive screening. Using BLASTp against the cucumber genome database, we identified PUB members containing conserved U-box domains and excluded proteins lacking this domain prior to subsequent analyses. Ultimately, 53 CsPUBs were systematically identified and designated as CsPUB01 to CsPUB53 according to their protein domain organization. The number of *PUB* genes in cucumber is fewer than that in Arabidopsis (64) and rice (77). Notably, 52 CsPUBs possessed intact U-box domains, while CsPUB07 exhibited partial deletion of N-terminal residues within the U-box domain (Figure 1).

To characterize the identified *CsPUBs*, several physicochemical parameters were analyzed, including gene identifier (ID), coding sequence (CDS) length, amino acid sequence length, molecular weight (MW), and theoretical isoelectric point (pI) (Table 1). The CsPUB proteins ranged from 235 to 1489 amino acid residues in length, with corresponding molecular weights spanning from 26 kDa (CsPUB48) to 166 kDa (CsPUB46). The theoretical pI values exhibited substantial variation, ranging from 4.43 (CsPUB52) to 9.04 (CsPUB36), with an average of 6.65.

**Figure 2:**
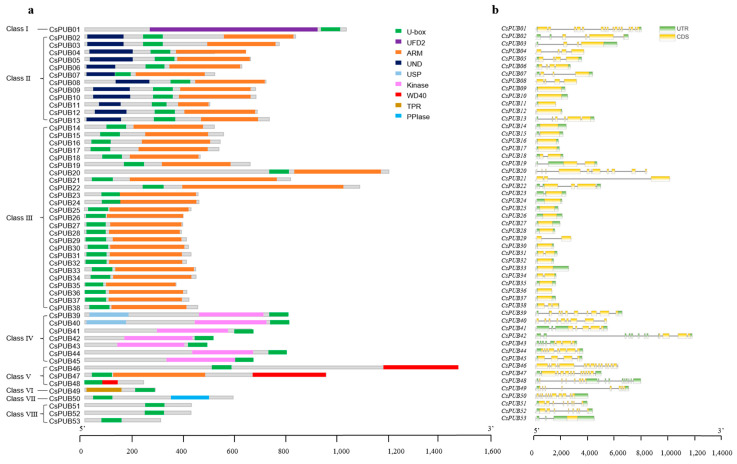
Analysis of conserved domains and gene structures of the PUB family in cucumber. (**a**) Nine conserved domains are delineated using different colors, with green representing the U-box domain. (**b**) The coding sequence region (CDS) and untranslated region (UTR) are represented in yellow and green, respectively, with black lines indicating intron regions.

The amino acid sequences of CsPUBs were utilized to query the protein families (Pfam) database, and the NCBI-CDD database was used to identify additional domains. Apart from the U-box domain, numerous other protein domains were found within these proteins, resembling those identified in Arabidopsis PUB proteins [17,33,34]. Based on their domain organization, CsPUBs have been classified into eight distinct classes (Figure 2).

Class I members exhibit similarity to UFD2 found in yeast. Only one member (*CsPUB01*) belongs to this class, akin to the classification observed in Arabidopsis [17]. Among plant PUBs, ARM repeat-containing proteins constitute the largest group due to the frequent combination of U-box and ARM repeats. Both Class II and Class III members possess ARM repeats, with an additional UND domain present in Class II members. Class III is the largest class with 25 members, followed by Class Ⅱ with 12 members. Members of Class IV are characterized by the presence of a USP domain and a kinase domain. Classes V, VI, and VII feature WD40, TPR, and PPlase domains, respectively, comprising 3, 1, and 1 member each. The residual CsPUBs fall into Class VIII (Figure 2a).

To elucidate the gene architecture of *CsPUB* genes, we retrieved exon–intron details for 53 *CsPUB* genes from the cucumber genome database using custom scripts. Subsequently, TBtools software was utilized to visualize these gene structures (Figure 2b). The number of exons within *CsPUB* genes exhibits significant variation from 1 to 17. Notably, *CsPUB48* possesses the highest exon count at 17, whereas 18 *PUB* genes consist of a solitary exon. Among the 25 class Ⅲ *CsPUB* genes, 20 contain either 0 or 1 intron, while all 10 class Ⅳ and class Ⅴ *CsPUB* genes have 7–17 exons, except for *CsPUB45*, which has 4. Additionally, both exon and intron lengths display diversity; for instance, numerous *CsPUBs* feature multiple small exons, yet *CsPUB21* and *CsPUB42* each harbor an exceptionally large exon. This intricate composition of *CsPUB* genes and their corresponding proteins underscores the complex evolution of *CsPUBs*.

### 2.2. Evolutionary Analysis of CsPUB Members

**Figure 3:**
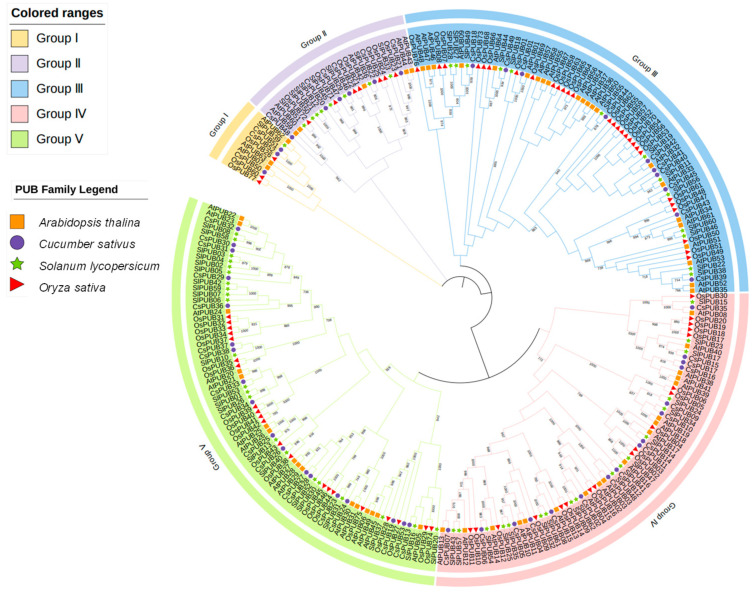
Evolutionary analysis of the PUB proteins in Arabidopsis, cucumber, rice and tomato.

To elucidate the evolutionary relationships among *CsPUB* genes and assess the evolutionary trajectory of this protein family, we employed ClustalW to conduct a multiple sequence alignment and then constructed a CsPUB proteins tree in Mega-7 based on the PUB proteins from cucumber (53 members), Arabidopsis (64 members), rice (77 members) and tomato (62 members) (Figure 3). According to the PUB proteins tree, CsPUBs are classified into five major subfamilies. Additionally, we constructed a separate evolutionary tree using highly conserved U-box domain sequences (Figure S1). However, the resulting evolutionary relationships markedly differed from those of the full-length CsPUB sequences, suggesting non-collinear evolution between the U-box domain and other regions.

### 2.3. Chromosome Localization and Collinearity Analysis of CsPUB Genes

**Figure 4:**
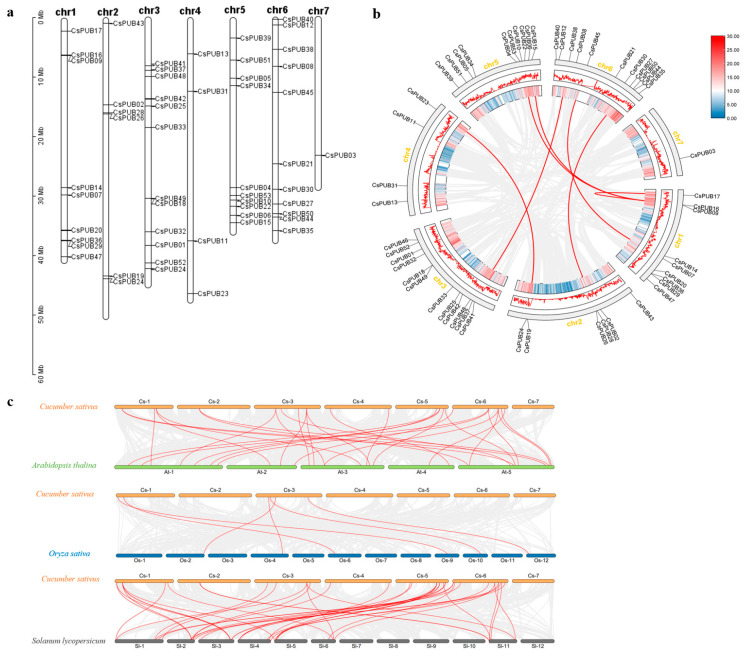
Chromosome analysis and collinearity analysis of the *CsPUB* family members. (**a**) Localization of *CsPUB* on chromosomes. The scale represents equal segments of cucumber chromosomes in megabases (Mb). (**b**) Collinearity analysis between *CsPUBs* in the whole cucumber genome. Red lines indicate genes with high homology, and the chromosome numbers are displayed on the outer side. Blue and red represent the gene density of the chromosomes, with the density increasing from blue to red. (**c**) Synteny analysis between cucumber *(C. sativus*) and Arabidopsis (*A. thaliana*), rice (*O. sativa*), or tomato (*S. lycopersicum*). Gray lines represent collinear relationships of all genes between the species pairs, while red lines indicate synteny among members of the *PUB* gene family.

The 53 *CsPUBs* exhibit an uneven distribution pattern across the genome, with no positive correlation between chromosome length and *CsPUB* gene number. As shown in Figure 4, among the seven cucumber chromosomes, only one *CsPUB* gene (*CsPUB03*) is located on Chr 7, whereas Chr 3 harbors the largest number of *CsPUBs* (12 genes), followed by Chr 6 with 11 genes. Chr 1, 2, 4, and 5 encode 9, 6, 4, and 10 *CsPUBs*, respectively. Notably, some *CsPUB* genes exhibit adjacent locations, such as *CsPUB09*-*CsPUB16*, *CsPUB26*-*CsPUB28*, and *CsPUB19*-*CsPUB24*. Among them, two pairs (*CsPUB29*-*CsPUB36* and *CsPUB18*-*CsPUB49*) display tandem repeats, suggesting that these tandemly duplicated genes are likely closely related (Figure 4a).

Collinearity analysis revealed high conservation at the nucleotide sequence level among *CsPUB* genes, with seven gene pairs identified (Figure 4b). These segmentally duplicated *CsPUB* gene pairs included: *CsPUB24/CsPUB23, CsPUB37/CsPUB38*, *CsPUB27/CsPUB28*, *CsPUB07/CsPUB45*, *CsPUB15/CsPUB16*, *CsPUB09/CsPUB10*, and *CsPUB16/CsPUB17*. These findings indicate that segmental duplication events contributed to the expansion of the *CsPUB* gene family.

To better understand the expansion patterns of *CsPUB* genes during evolution, we performed comparative synteny analyses between cucumber and three reference species: Arabidopsis (*Arabidopsis thaliana*), rice (*Oryza sativa*), and tomato (*Solanum lycopersicum*). A total of 77 syntenic gene pairs were identified involving *CsPUB* genes, with 30 pairs between cucumber and Arabidopsis, 6 pairs between cucumber and rice, and 41 pairs between cucumber and tomato (Figure 4c). These results reveal closer evolutionary relationships between cucumber *PUB* genes and those in tomato, while demonstrating more distant syntenic conservation with monocotyledonous species rice.

### 2.4. Cis-Acting Elements Analysis of the Promoter of CsPUBs

**Figure 5:**
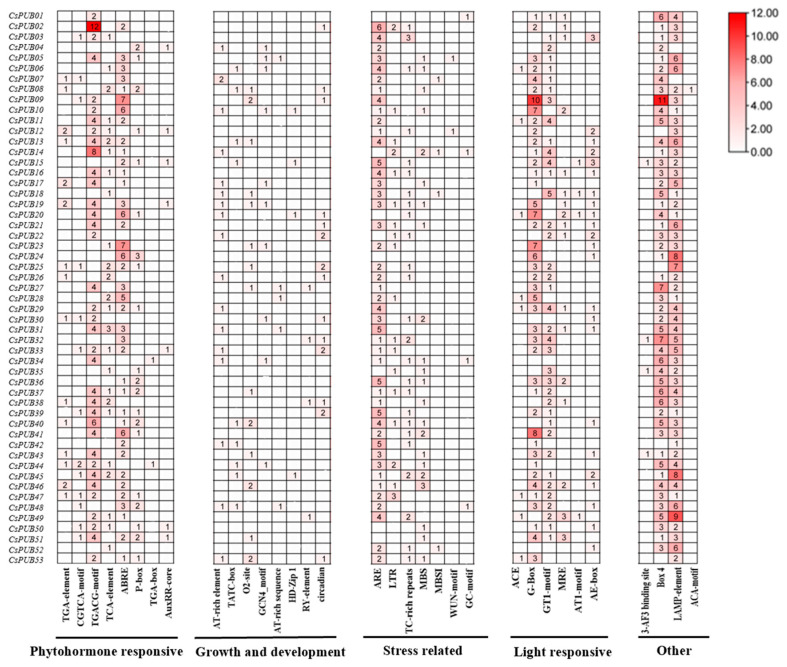
Analysis of *cis*-acting elements in *CsPUBs*. The *cis*-acting element analysis of *CsPUBs* is mainly divided into five major categories: plant hormone responsiveness, growth and development-related, biotic and abiotic stress-related, light responsiveness, and some other elements. The numerical values in the upper right corner indicate statistical counts of *cis*-elements, with color intensity scaled proportionally to the count magnitude (darker hues = higher counts).

To investigate *CsPUB* gene expression regulation and their potential function in plant development and stress response, the *cis*-elements within the 2000 bp promoter regions upstream of the start codon of these genes were analyzed. The PlantCARE database was employed to dig out the possible *cis*-elements. A total of 33 *cis*-regulatory elements were identified in the promoter region of *CsPUBs* (Figure 5 and Figure S2). These *cis*-regulatory elements can be classified into five categories that are associated with phytohormones, plant growth and development, biotic and abiotic stress, light, and other factors, respectively (Figure S2). The *cis*-elements that are associated with phytohormone response, such as TGACG motif/CGTCA motif, ABRE motif, P-box, AuxRR-core, TGA motif, and TCA element, are largely enriched in the promoter of the *CsPUB* genes. The biotic and abiotic stress response elements, including ARE, MBS, WUN motif, TC-rich repeats, and LTR, are also enriched in these genes. Considering the reported *PUBs* function in other plant species, the *CsPUBs* are also likely to play crucial roles in plant disease resistance and abiotic stress adaptation. *Cis*-acting elements associated with plant growth and development (e.g., TATC-box, GCN4-motif, and AT-rich element), as well as light-responsive elements (e.g., G-box, ACE, and AT1-motif), were also identified in the *CsPUB* promoters, suggesting that *CsPUBs* are likely involved in regulating diverse biological processes.

### 2.5. Expression Analysis of CsPUBs

**Figure 6:**
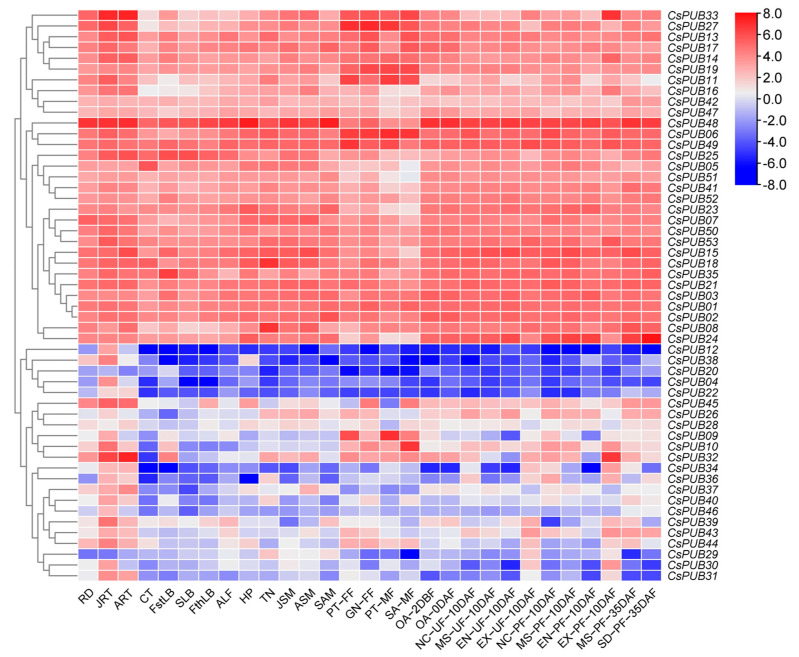
The heatmap depicts the expression patterns of 53 *CsPUB* genes in various plant tissues of cucumber. Red indicates upregulation of gene expression, while blue indicates downregulation of gene expression. The different tissue organs are as follows: radicle (RD), juvenile root (JRT), adult root, cotyledon (CT), first true leaf blade (FstLB), second true leaf blade (SLB), fourth true leaf blade (FthLB), adult leaf (ALF), hypocotyl (HP), tendril (TN), juvenile stem (JSM), adult stem (ASM), shoot apical meristem (SAM), petals from female flowers (PT-FF), gynoecium from female flowers (GN-FF), petals from male flowers (PT-MF), stamens from male flowers (SA-MF), ovary at two days prior to flowering (OA-2DBF), ovary on the day of flowering (OA-0DAF), neck sampled from unpollinated fruits at 10 days after flowering (NC-UF-10DAF), mesocarp sampled from unpollinated fruits at 10 days after flowering (MS-UF-10DAF), endocarp sampled from unpollinated fruits at 10 days after flowering (EN-UF-10DAF), exocarp sampled from unpollinated fruits at 10 days after flowering (EX-UF-10DAF), neck sampled from pollinated fruits at 10 days after flowering (NC-PF-10DAF), mesocarp sampled from pollinated fruits at 10 days after flowering (MS-PF-10DAF), endocarp sampled from pollinated fruits at 10 days after flowering (EN-PF-10DAF), exocarp sampled from pollinated fruits at 10 days after flowering (EX-PF-10DAF), mesocarp sampled from unpollinated fruits at 35 days after flowering (MS-UF-35DAF), and seeds sampled from unpollinated fruits at 35 days after flowering (SD-UF-35DAF).

To further study the potential functions of *CsPUB* genes, we analyzed their spatiotemporal expression patterns by collecting and examining cucumber RNA-seq data. As shown in Figure 6, based on their expression profiles across various tissues and developmental stages, the *CsPUB* genes were categorized into three distinct groups. Group 1 genes (including *CsPUB38*, *CsPUB12*, *CsPUB20*, *CsPUB04*, *CsPUB22*, *CsPUB29*, *CsPUB30*, *CsPUB31*, *CsPUB34*, *CsPUB36*, *CsPUB40*, and *CsPUB46*) exhibited low expression levels in most tissues examined. Nevertheless, they may show a higher expression level in some specific tissues. Group 3 genes exhibited high expression in most or all of the tissues examined; however, for group 2 genes, their expression levels varied significantly across different tissues.

**Figure 7:**
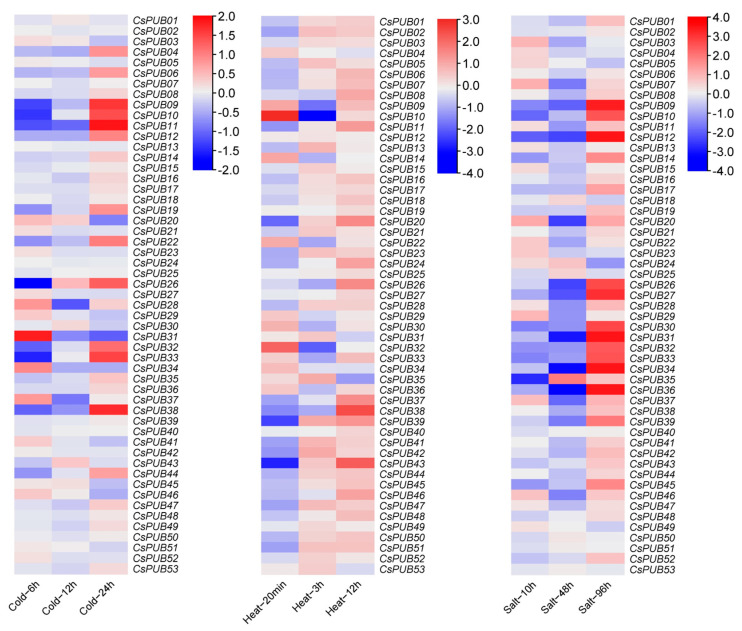
Heatmap of expression patterns for 53 *CsPUB* under different abiotic stress treatments (cold, heat, and salt). Red indicates upregulation of gene expression, while blue indicates downregulation of gene expression.

**Figure 8:**
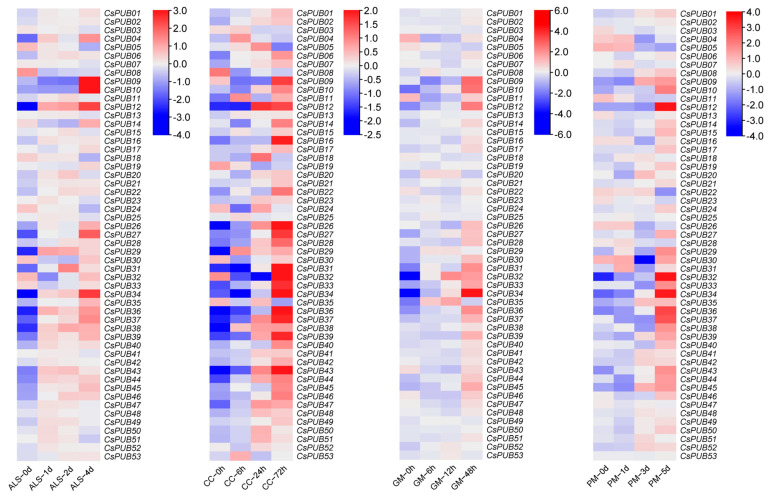
Heatmap of expression patterns for 53 *CsPUB* genes under different pathogen treatments. The four panels illustrate expression level changes of the 53 *CsPUB* genes at distinct time points following inoculation with: *Pseudomonas syringae* (angular leaf spot, ALS), *Cladosporium cucumerium* (CC), *Botrytis cinerea* (gray mold, GM), and *Podosphaera xanthii* (powdery mildew, PM). Gene relative expression intensity is scaled from high (red) to low (blue).

In model plants, *PUB* genes are extensively involved in defense responses and tolerance to abiotic stress. Given the enrichment of *cis*-elements associated with biotic and abiotic stress responses in *CsPUB* promoters, we analyzed the expression levels of *CsPUB* genes under diverse abiotic and biotic stress conditions using publicly available cucumber transcriptomic datasets [32]. The expression of *CsPUB* genes was examined following cold, heat, and salt treatments. As shown in Figure 7, multiple *CsPUB* genes exhibited differential expression under these stress conditions. Additionally, we analyzed *CsPUB* expression in response to four pathogens causing angular leaf spot (*Pseudomonas syringae* pv. *lachrymans*, *Psl*), scab disease (*Cladosporium cucumerium*), gray mold, and powdery mildew. As depicted in Figure 8, many *CsPUB* genes showed differential expression upon infection with *Psl* and other pathogens. Notably, most *CsPUB* genes were upregulated during pathogen infection. For example, *CsPUB44* expression peaked at day 3 or 4 post-inoculation.

**Figure 9:**
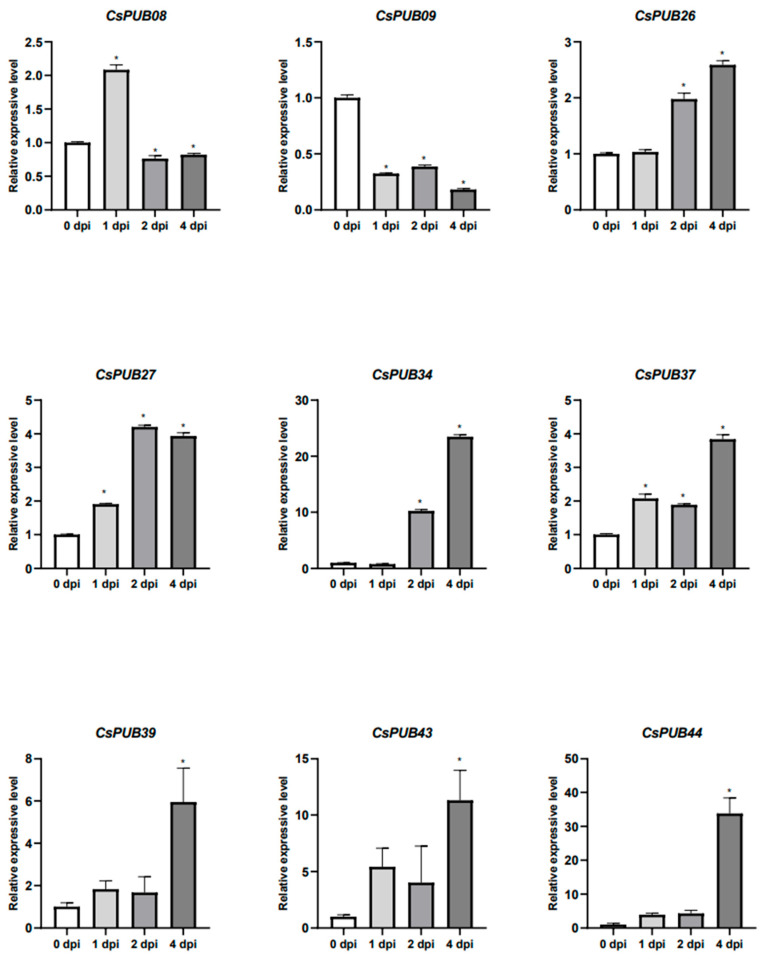
Expression analysis of CsPUB08, CsPUB09, CsPUB26, CsPUB27, CsPUB34, CsPUB37, *CsPUB39*, *CsPUB43*, and *CsPUB44*, after *Pseudomonas syringae* pv. *lachrymans* (*Psl*) treatment. Four-week-old cucumber seedlings were treated with *Psl*, and leaves were collected at 0, 1, 2, and 4 days after inoculation (dpi). *UBQ* was used as the internal reference gene to quantitatively analyze the transcription level of *CsPUB* through RT-qPCR. Significant differences between 0 dpi and other time points are indicated by asterisks (* *p* < 0.05).

To further validate the role of *CsPUB* genes in defense responses, cucumber seedlings were treated with *Pseudomonas syringae* pv. *lachrymans*. The expression patterns of *CsPUB08*, *CsPUB09*, *CsPUB26*, *CsPUB27*, *CsPUB34*, *CsPUB37*, *CsPUB39*, *CsPUB43*, and *CsPUB44* were examined using quantitative reverse transcription PCR (RT-qPCR) following pathogen inoculation. All tested *CsPUB* genes showed significantly increased expression after pathogen challenge, strongly suggesting their potential roles in plant immunity (Figure 9).

### 2.6. Subcellular Localization Analysis

**Figure 10:**
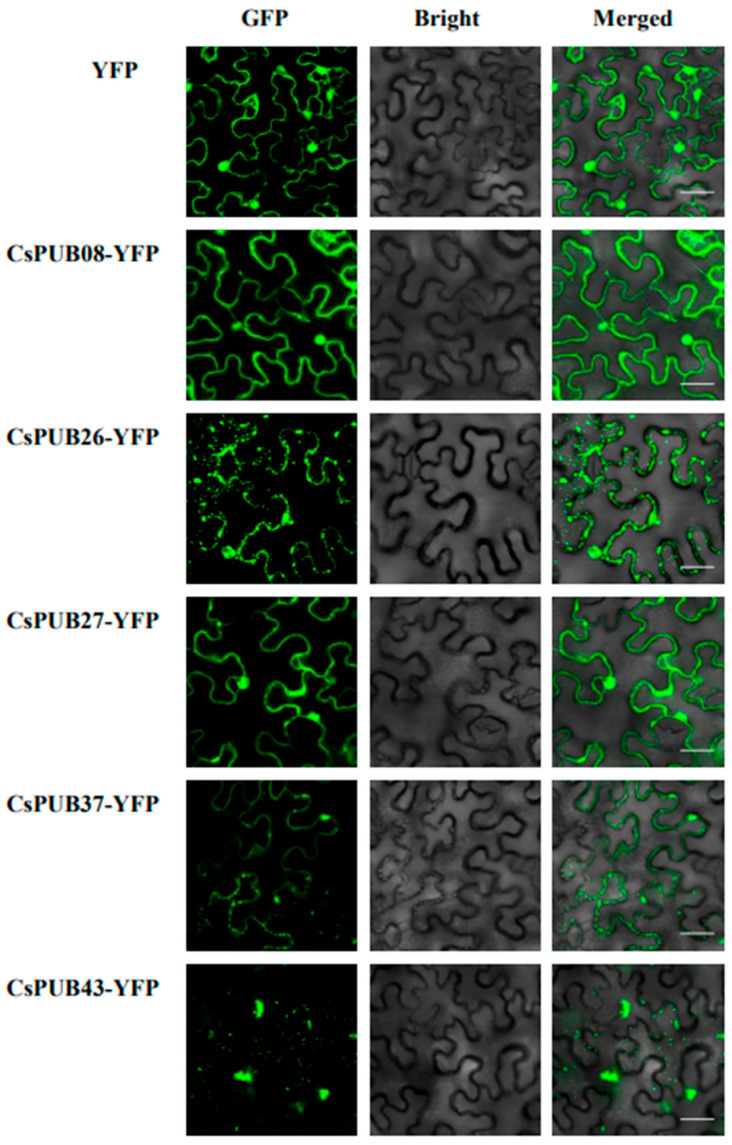
Subcellular localization analysis of CsPUBs. Fluorescent signals were detected by confocal laser scanning microscopy in *Nicotiana benthamiana* cells expressing CsPUB08-YFP, CsPUB26-YFP, CsPUB27-YFP, CsPUB37-YFP, and CsPUB43-YFP, 60 h after agrobacterial infiltration. The YFP empty vector was used as the control. Bars = 40 μm.

Five pathogen-induced CsPUB proteins from the three major classes (CsPUB08, 26, 27, 37, 43) were selected for subcellular localization analysis. CsPUB08 and CsPUB27 localized to both the nucleus and cytoplasm, whereas CsPUB26 and CsPUB37 were predominantly distributed in the cytoplasm. Intriguingly, CsPUB26, CsPUB37, and CsPUB43 exhibited distinct punctate structures that did not colocalize with the nucleus. These puncta varied in size in different CsPUBs. CsPUB26 accumulated in small punctate structures, while CsPUB37 and CsPUB39 formed larger punctate structures (Figure 10).

## 3. Discussion

The U-box domain is widely present in eukaryotes. Compared to yeast and humans, whose genomes encode only a few U-box-containing proteins, plant genomes have undergone significant expansion in U-box protein-encoding genes [17]. To date, *PUB* genes have been identified in the genome of dozens of plant species, including *Arabidopsis thaliana* (64), *Oryza sativa* (77), *Cucumis sativus* (53), *Glycine max* (121), *Nicotiana tabacum* (116), and *Triticum aestivum* (213), all of which encode over 50 *PUB* genes [17,34]. Generally, polyploid plants tend to harbor more *PUB* genes than diploid species. However, this does not imply that larger genomes necessarily encode more *PUBs*. For instance, although the cucumber genome (322 Mb) is significantly larger than that of Arabidopsis (125 Mb) [32], cucumber contains fewer *PUB* genes. This likely results from significantly fewer gene duplication events in *CsPUBs*, as demonstrated by the identification of merely seven collinear gene pairs, substantially fewer than reported in other eudicots such as tomato (*S. lycopersicum*) [35]. Notably, tomato *PUB* genes exhibit closer phylogenetic relationships with *CsPUBs* than those observed in either Arabidopsis or rice (*O. sativa*).

The U-box, a highly conserved domain, mediates interactions between plant U-box proteins (PUBs) and E2 ubiquitin-conjugating enzymes (E2s), as well as the formation of dimers/oligomers, with deletions or mutations of its conserved residues abolishing the ability of PUBs to stimulate E2-dependent ubiquitination [17,34]. For example, the conserved cysteine 239 and glycine 255 in AtPUB4 [19,36], cysteine 13, valine 24, and tryptophan 40 in AtPUB22 [37,38,39], and cysteine 262 and tryptophan 289 in AtPUB13 [23], cysteine 281 of OsPUB2, and cysteine of OsPUB3 [40], are critical for maintaining E3 ligase activity and functionality. Mutations in these residues disrupt PUB-mediated processes, such as drought stress response, low temperature response, and immune response. These residues are also highly conserved in CsPUB proteins.

The U-box is usually combined with other domains, including ARM repeats, UFD2, TPR, kinase, USP, PPIase, and so on. The most common arrangement is the U-box domain–ARM repeats domain combination. The PUB proteins are usually classified into several groups according to the arrangement of the protein domains. Most of the domains present in PUB in plant species like Arabidopsis and rice are also found in CsPUBs [17]. The ARM repeats domain normally mediates protein–protein interaction and is believed to determine the substrate specificity during ubiquitination [34]. Similar to that in Arabidopsis, most CsPUBs (38 out of 53) contain the ARM repeat domain. Nevertheless, there are also some distinct differences between PUBs from Arabidopsis and cucumber. The MIF4G domain containing PUB has not been identified in the latter. Furthermore, the USP–kinase combination is also lacking in cucumber PUB proteins. These indicate the complex evolution of plant PUB proteins.

In our study, phytohormone response elements and stress-related elements are enriched in the promoter regions of *CsPUBs*. Transcriptome and quantitative PCR analyses demonstrate that various *CsPUBs* respond to abiotic and biotic stresses, suggesting their possible functional roles in phytohormone signaling and stress adaptation. Indeed, *PUB* family genes are extensively documented to regulate both biotic and abiotic stress responses as well as hormone pathways: for instance, *OsPUB16* suppresses Abscisic acid (ABA) and JA biosynthesis to reduce drought tolerance [41], SlPUB22 targets the JA signaling repressor jasmonate zinc finger inflorescence meristem-domain 4 (JAZ4) for degradation to enhance jasmonate responses [41], and AtPUB35 ubiquitinates the ABA-negative regulator ABA-insensitive 5 (ABI5) to modulate ABA signaling [42]. In biotic stress, *AtPUB2/4*, *AtPUB25/26*, *OsPUB44*, and *VsPUB26* positively regulate disease resistance [21,24,28,29,43], whereas *AtPUB12/13*, *AtPUB22/23/24*, *OsPUB9*, and *OsPUB12* act as negative immune regulators [23,27,44,45]. For abiotic stress, *PUBs* mediate tolerance to temperature extremes (e.g., *AtPUB25/26* and *MdPUB23*) [46,47], drought (e.g., *OsPUB75* and *OsPUB16*) [41,48], osmotic stress (e.g., *AtPUB44*) [49], and oxidative stress (e.g., *AtPUB2*) [50]. Given the conserved *cis*-element signatures, functional parallels across species, and the critical roles of *PUBs* in hormone crosstalk (e.g., JA, ABA) and stress signaling networks, *CsPUBs* are likely to function analogously in regulating phytohormone signaling and stress responses within their native biological contexts.

PUB proteins are localized to various organelles to regulate distinct cellular activities. For example, Modifier of *snc1*, 4 (MOS4)-associated complex 3A (MAC3A/AtPUB59) and MAC3B/AtPUB60 localize to the nucleus and are required for proper splicing of plant resistance genes, thereby conferring resistance to pathogens [51]. AtPUB22 and AtPUB23 are exclusively cytosolic [52], while OsPUB67 is ubiquitously distributed in the nucleus, cytosol, and membrane systems [53]. In Arabidopsis, PUB9 localizes to punctate structures distinct from known organelles. Upon coexpression with the kinase Arabidopsis receptor kinase2 (ARK2), PUB9 colocalizes with the autophagosomal marker autophagy-related 8 (ATG8) [54]. When coexpressed with E2 components, including OsUBC18/25/27/29, OsPUB67 was also found to localize in the punctate structures [53]. In our study, CsPUB25, OsPUB39, and OsPUB50 exhibit AtPUB9- and OsPUB67-like punctate localization, suggesting this pattern is likely common among CsPUBs. Elucidating the precise organellar identity of these punctate structures will shed light on PUB-mediated cellular responses not only in cucumber but across plant species.

This study conducts the first comprehensive genomic and functional characterization of the PUB E3 ubiquitin ligase family in *C. sativus*, systematically analyzing their evolutionary relationships, gene structures, conserved domains, chromosomal distributions, collinearity and evolutionary relationship, expression patterns across different tissues and stress conditions, and subcellular localization patterns. Through genome-wide screening, we identified 53 *CsPUB* genes exhibiting both conserved features and species-specific characteristics. Bioinformatics analyses revealed that many *CsPUBs* contain stress-responsive *cis*-elements and show differential expression under abiotic and biotic stresses, suggesting their potential roles in stress adaptation. While current technical limitations in cucumber transformation hinder direct functional validation, the rapid development of genetic transformation, CRISPR-based genome editing, and transient expression systems in cucurbits will enable future investigations through targeted genetic manipulations. These findings establish an important foundation for understanding PUB-mediated regulatory networks in cucumber and provide valuable potential genetic targets for improving stress resistance in cucurbit crops through molecular breeding approaches.

## 4. Materials and Methods

### 4.1. Plant Materials and Growth Conditions

The cucumber (*Cucumis sativus*) inbred line 9930 was used in this study. Cucumber plants were cultivated for seed production in a greenhouse at 25 °C with a 16-h light/8-h dark photoperiod. For pathogens treatment, the seedlings at the two-cotyledon stage, initially grown in 9 cm × 9 cm pots, were transplanted into 2-gallon pots containing soil. Plants were maintained in growth chambers at 25 °C with a 16-h light/8-h dark photoperiod. *Nicotiana benthamiana* plants were grown under the same conditions for subsequent experiments.

### 4.2. Identification of Plant U-Boxes and Construction of Evolutionary Tree and Protein Information Analysis

To systematically identify U-box genes in the cucumber genome, the following bioinformatics pipeline was implemented. The most recent cucumber genome assembly (version 4.0) was retrieved from the Cucumber Genome Database (Cucumber V4; http://www.cucumberdb.com/#/download, last accessed: 5 November 2024). The Hidden Markov Model (HMM) profile corresponding to the U-box domain (Pfam: PF04564) was acquired from the InterPro database (https://www.ebi.ac.uk/interpro/, last accessed: 8 November 2024). Initial candidate *CsPUB* genes were identified through HMMER searches (E-value ≤ 1e-5) executed in TBtools software [55].

Complementary identification was performed using BLASTp (Tbtools, v2.310) analysis (E-value ≤ 1e-10) with 64 experimentally validated Arabidopsis U-box protein sequences (TAIR10; https://www.arabidopsis.org/, last accessed: 15 October 2024) as queries against the cucumber proteome. Consensus candidates from both HMM and BLAST approaches were determined through Venn diagram analysis using TBtools’ visualization module. Domain validation was conducted through InterProScan (https://www.ebi.ac.uk/interpro/search/sequence/, last accessed: 20 November 2024) and NCBI’s Conserved Domain Database (CDD; https://www.ncbi.nlm.nih.gov/Structure/cdd/wrpsb.cgi, last accessed: 25 November 2024). The PUB proteins tree was constructed using MEGA7 with the following parameters: ClustalW alignment, neighbor-joining method, and 1000 bootstrap replicates [56]. Visual refinement of the evolutionary tree was performed using the iTOL platform (https://itol.embl.de/, last accessed: 28 May 2025).

### 4.3. Chromosome Localization and Collinearity Analysis

The genomic sequences of *CsPUBs* were extracted from the cucumber genome GFF3 annotation file and subjected to local visualization through TBtools software. Intra-species collinearity analysis in cucumber was conducted using the Advanced Circos module of TBtools for chromosomal distribution mapping. For inter-species collinearity analysis among cucumber, Arabidopsis, and rice, genome annotation files were retrieved from TAIR 10 and Plants Ensembl databases (https://plants.ensembl.org/index.html, last accessed: 24 October 2024), respectively. The Multiple Synteny Plot function in TBtools was subsequently employed for comparative visualization of evolutionary relationships across these species.

### 4.4. Gene Structure, Conserved Domain, and Cis-Acting Element Analysis

This study employed the cucumber genome GFF3 annotation file to extract coding sequences (CDS) and untranslated regions (UTRs) using TBtools bioinformatics software, followed by a comprehensive visualization analysis of gene structures for *CsPUB* gene family members. For identifying conserved domains in CsPUB proteins, a dual-strategy bioinformatics approach was implemented: (1) motif prediction through the Motif Elicitation module of the MEME Suite platform (https://meme-suite.org/meme/tools/meme, last accessed: 6 November 2024), and (2) functional domain annotation using the CDD website from NCBI. The analytical results were cross-validated through both methods, and final integrated schematic diagrams of CsPUB conserved domains were constructed using professional graphical software.

Furthermore, promoter sequences spanning 2000 bp upstream of the initiation codon ATG were retrieved via TBtools. Systematic prediction of *cis*-acting elements was performed using the PlantCare database (https://bioinformatics.psb.ugent.be/webtools/plantcare/html/, last accessed: 10 December 2024), with particular emphasis on identifying stress-responsive regulatory elements.

### 4.5. Pathogen Treatment

To investigate pathogen-induced expression patterns of *PUB* genes, cucumber seedlings were inoculated with *Pseudomonas syringae* pv. *lachrymans* (*Psl)* through spray inoculation. Environmental control: Constant temperature (25 ± 0.5 °C) was maintained. Post-inoculation humidity was elevated to >85% to facilitate infection establishment, followed by restoration to 50 ± 5% RH at 48 hpi. Healthy seedlings at the two-true-leaf stage were treated with bacterial suspensions (10^6^ cfu/mL). Samples were collected before inoculation (0 dpi) as controls, and at 1, 2, 4 days post-inoculation (dpi) for expression profiling via RT-qPCR. Three biological replicates were performed for each treatment group. For each biological replicate, two 1.5-cm-diameter leaf discs (from a single leaf) were collected into a sterile 1.5-mL Eppendorf tube. The samples were immediately flash-frozen in liquid nitrogen and stored at −80 °C until further processing.

### 4.6. Gene Expression Analysis

RNA-seq datasets encompassing diverse tissues, biotic stresses, and abiotic stresses were retrieved from the public cucumber genome database (Cucumber V4; http://www.cucumberdb.com/#/download, last accessed: 5 November 2024). Expression heatmaps were generated using TBtools v2.225 through sequential steps: log_2_-transformation of expression values, data normalization, and hierarchical clustering, with expression levels represented by a “Blue-White-Red” color gradient (low to high).

To investigate the expression regulation patterns of the *CsPUB* genes under pathogen infection, total RNA was extracted using the FastPure^®^ Plant Total RNA Isolation Kit (Vazyme, Nanjing, China). Uniformly processed 0.045 g of plant tissue was used for RNA extraction across all experiments. Subsequently, 2 μg of total RNA was subjected to reverse transcription for each sample. Subsequently, the extracted RNA was reverse-transcribed into cDNA using the HiScript^®^ III 1st Strand cDNA Synthesis Kit from the same manufacturer. Quantitative PCR analysis was executed on the 7500 Real-Time PCR System (BIO-RAD, Hercules, CA, USA) with the AceQ^®^ Universal SYBR qPCR Master Mix (Vazyme, Nanjing, China). Three biological replicates with triplicate technical repetitions were conducted for each *CsPUB* gene and the reference gene *UBQ* (CsaV4_5G002584). Relative expression levels were calculated using the 2^−ΔΔCt^ method [57]. Primer sequences are provided in Supplementary Table S1.

### 4.7. Subcellular Localization

Gene-specific primers were designed to amplify the full-length coding sequences (stop codon excluded) of CsPUB37, CsPUB8, CsPUB26, CsPUB27, and CsPUB43. These sequences were cloned into the pEarleyGate-301UBQYFP vector and subsequently introduced into *Agrobacterium tumefaciens* strain GV3101. Bacterial suspensions were infiltrated into mature leaves of *N. benthamiana* plants. YFP fluorescence signals were captured 60 h post-infiltration using a confocal laser scanning microscope (Zeiss LSM880, Jena, Germany) with excitation at 514 nm. Primer sequences are listed in Supplementary Table S1.

## 5. Conclusions

This study provides a comprehensive genomic and functional characterization of the PUB E3 ligases in *C. sativus* and novel insights into their roles in stress responses. Systematic genome-wide screening led to the identification of 53 *PUB* genes in the cucumber genome. Conserved domain analysis, gene structure examination, evolutionary assessment, and chromosomal localization revealed both the conservation and diversity of these genes. *Cis*-acting element prediction and gene expression pattern analysis indicated that many members exhibit tissue-specific expression and respond to both abiotic and biotic stresses. Subcellular localization analysis demonstrated diverse protein localization patterns. These findings reveal the functional diversity of PUB proteins in cucumber and provide potential gene targets to develop stress-tolerant/resistant cucumber resources.

## Figures and Tables

**Table 1:** Information on *PUB* Family Genes in Cucumber.

Gene ID	Gene Name	AA	MW	pI	Chr	Location	SD
CsaV4_3G004078	*CsPUB01*	1043	117,952.08	5.4	3	38,288,116–38,297,099	+
CsaV4_2G001478	*CsPUB02*	1142	131,432.04	6.37	2	14,584,660–14,591,279	−
CsaV4_7G001821	*CsPUB03*	776	84,567.83	6.1	7	23,414,401–23,421,303	−
CsaV4_5G002287	*CsPUB04*	642	17,463.87	4.95	5	28,595,090–28,597,391	+
CsaV4_5G001121	*CsPUB05*	661	72,164.67	6.32	5	10,256,146–10,260,002	−
CsaV4_5G003054	*CsPUB06*	189	20,192.19	6.34	5	33,278,778–33,279,989	−
CsaV4_1G002812	*CsPUB07*	519	56,015.17	6.65	1	29,784,570–29,789,371	−
CsaV4_6G001024	*CsPUB08*	292	31,777.82	6.01	6	8,320,818–8,324,444	−
CsaV4_1G000903	*CsPUB09*	681	75,410.55	8.32	1	6,312,230–6,314,661	−
CsaV4_5G002605	*CsPUB10*	683	76,794.22	7.42	5	30,687,010–30,689,661	−
CsaV4_4G002233	*CsPUB11*	498	55,784.36	6.3	4	37,600,005–37,601,623	+
CsaV4_6G000201	*CsPUB12*	688	75,882.62	7.85	6	1,364,117–1,366,286	+
CsaV4_4G000578	*CsPUB13*	738	82,591.93	6.04	4	6,139,853–6,144,787	+
CsaV4_1G002691	*CsPUB14*	517	55,681.77	6.81	1	28,557,228–28,559,764	−
CsaV4_5G003208	*CsPUB15*	554	60,418.4	7.56	5	34,474,333–34,476,587	−
CsaV4_1G000891	*CsPUB16*	540	59,407.43	6.63	1	6,255,763–6,257,621	+
CsaV4_1G000189	*CsPUB17*	452	49,441.99	5.65	1	2,219,249–2,221,155	+
CsaV4_3G002876	*CsPUB18*	291	32,302.81	8.79	3	30,441,628–30,442,788	−
CsaV4_2G002560	*CsPUB19*	659	72,594.83	5.16	2	43,412,255–43,417,433	−
CsaV4_1G003549	*CsPUB20*	1213	137,319.25	6.23	1	35,703,460–35,712,913	+
CsaV4_6G002143	*CsPUB21*	495	53,838.87	5.11	6	24,740,406–24,742,324	−
CsaV4_5G002792	*CsPUB22*	1014	113,306.44	5.35	5	31,741,490–31,746,985	+
CsaV4_4G003100	*CsPUB23*	453	49,087.09	5.83	4	46,390,827–46,393,341	+
CsaV4_2G002621	*CsPUB24*	455	49,490.86	6.67	2	43,808,284–43,810,452	−
CsaV4_3G001913	*CsPUB25*	425	46,364.36	5.97	3	14,930,324–14,932,165	−
CsaV4_2G001668	*CsPUB26*	399	44,849.97	5.85	2	16,182,318–16,184,507	−
CsaV4_6G003001	*CsPUB27*	404	44,791.15	8.2	6	31,428,505–31,430,499	−
CsaV4_2G001635	*CsPUB28*	387	43,290.96	7.91	2	15,924,847–15,926,401	−
CsaV4_1G003701	*CsPUB29*	265	29,234.28	6.14	1	37,471,774–37,474,930	−
CsaV4_6G002670	*CsPUB30*	414	45,873.46	8.67	6	29,000,714–29,002,167	−
CsaV4_4G001209	*CsPUB31*	424	46,727.27	8.96	4	12,441,210–12,442,959	−
CsaV4_5G001204	*CsPUB34*	444	49,923.81	8.17	5	11,491,336–11,492,993	+
CsaV4_3G003735	*CsPUB32*	406	44,468.67	9.08	3	36,008,291–36,009,734	−
CsaV4_3G002201	*CsPUB33*	442	49,149.68	8.4	3	18,505,395–18,508,135	−
CsaV4_6G003794	*CsPUB35*	365	39,439.79	8.5	6	35,988,799–35,990,428	−
CsaV4_1G003699	*CsPUB36*	408	45,751.58	9.04	1	37,393,795–37,395,087	+
CsaV4_3G001063	*CsPUB37*	415	46,407.31	6.91	3	8,949,370–8,950,992	−
CsaV4_6G000661	*CsPUB38*	451	50,629.08	8.33	6	5,445,539–5,447,447	−
CsaV4_5G000484	*CsPUB39*	808	90,431.07	8.09	5	3,491,174–3,498,511	+
CsaV4_6G000061	*CsPUB40*	813	91,533.06	5.66	6	406,116–412,114	+
CsaV4_3G000934	*CsPUB41*	669	75,495.52	5.72	3	8,154,010–8,160,068	−
CsaV4_3G001789	*CsPUB42*	510	58,056.97	6.07	3	13,700,345–13,713,702	+
CsaV4_2G000042	*CsPUB43*	489	55,675.74	5.98	2	941,836–945,270	−
CsaV4_6G003328	*CsPUB44*	806	91,648.28	5.72	6	33,673,401–33,677,361	+
CsaV4_6G001517	*CsPUB45*	671	75,819.23	5.67	6	12,689,616–12,693,513	−
CsaV4_3G004576	*CsPUB46*	1489	165,762.75	5.84	3	42,249,414–42,256,392	−
CsaV4_1G003910	*CsPUB47*	962	106,853.78	5.24	1	40,158,873–40,164,408	−
CsaV4_3G001224	*CsPUB48*	235	26,027.79	6.72	3	9,905,953–9,914,891	−
CsaV4_3G002875	*CsPUB49*	281	32,050.38	5.41	3	30,429,405–30,437,301	+
CsaV4_6G003237	*CsPUB50*	592	65,020.26	8.16	6	33,109,149–33,113,551	−
CsaV4_5G000913	*CsPUB51*	425	47,216.88	5.04	5	7,225,532–7,229,873	−
CsaV4_3G004442	*CsPUB52*	308	33,595.38	4.43	3	41,242,520–41,247,309	−
CsaV4_5G002475	*CsPUB53*	303	33,708.96	4.77	5	29,913,450–29,918,387	+

Note: AA, length of amino acid sequence; MW, molecular weight; pI, isoelectric point; SD, strand direction.

## Data Availability

Data are contained within the article and supplementary materials.

## Supplementary Materials

The following supporting information can be downloaded at: https://www.mdpi.com/article/10.3390/plants14121801/s1, Figure S1: Evolutionary analysis of the U-box in CsPUBs; Figure S2: Statistical analysis of the number of *cis*-acting elements of CsPUBs; Table S1: List of primers used in this study.
