# Supplementary material for: Genome-Wide Identification, Characterization, and Expression Analysis of the U-Box Gene Family in Cucumber (Cucumis sativus)
Source: Plants (Basel). 2025 Jun 12;14(12):1801. doi: 10.3390/plants14121801 (PMC12196615; doi:10.3390/plants14121801)
Supplement: Supplementary file 1 [file plants-14-01801-s001.zip › Supplement Table.pdf]

Table S1. List of primers used in this study

**Primers for RT-qPCR**		
CsPUB08-Q-F	5'-GACGGGACTCCGAAGAACAAG-3'	160 bp
CsPUB08-Q-R	5'-AGGTTGCTTTTCTCTGGGCTC-3'	
CsPUB09-Q-F	5'-AGAGCTCGTTTGATTTAGTTTGG-3'	174 bp
CsPUB09-Q-R	5'-ACAGATAACCAATACACATTGACG-3'	
CsPUB26-Q-F	5'-ACTCGGAAACACAACCGAGC-3'	151 bp
CsPUB26-Q-R	5'-TCCAGTAACTGCACCTTCCAC-3'	
CsPUB27-Q-F	5'-TCCGATTCCCTTCACCAACG-3'	191 bp
CsPUB27-Q-R	5'-TCGACAAGTAGGGGGAGGAG-3'	
CsPUB34-Q-F	5'-TCGAGTTGACGATTCCGACC-3'	124 bp
CsPUB34-Q-R	5'-GTTGGTTGGTGAAAGGGCAG-3'	
CsPUB37-Q-F	5'-GACGATGCAGACACTCGCTG-3'	150 bp
CsPUB37-Q-R	5'-ACAGTAGCAAGAAGCGACGAG-3'	
CsPUB39-Q-F	5'-GGAGGTGACGGCAGATCAAG-3'	199 bp
CsPUB39-Q-R	5'-TCCCATTGGAGTTGGGACAC-3'	
CsPUB43-Q-F	5'-CAATGGGGGATCTTGTGGCTG-3'	187 bp
CsPUB43-Q-R	5'-CTCAAAAGTGCAGTGGCAGG-3'	
CsPUB44-Q-F	5'-TCACACTCCATTTGGGAAGC-3'	135 bp
CsPUB44-Q-R	5'-TCTGCTCTTACCTTGCCACAG-3'	
CsUBQ-Q-F	5'-CACCAAGCCCAAGAAGATC-3'	222 bp
CsUBQ-Q-R	5'-TAAACCTAATCACCACCAGC-3'	
**Primes for subcellular localization**		
CsPUB8-YFP-F	5'-ctgagtttttctgattaacagATGGGAGACACTCCATCTGATC-3'	915 bp
CsPUB8-YFP-R	5'-gcccttgctcaccatggatccGGGAATGTGTTCACACTTGCTC-3'	
CsPUB26-YFP-F	5'-ctgagtttttctgattaacagATGAAAGAAGCTGATGATCATGAG-3'	1236 bp
CsPUB26-YFP-R	5'-gcccttgctcaccatggatccCATATTCCTTTTGTCCTCAACCC-3'	
CsPUB27-YFP-F	5'-ctgagtttttctgattaacagATGGTGAAAGATGACTTATGTATTAC-3'	1251 bp
CsPUB27-YFP-R	5'-gcccttgctcaccatggatccACATGGCATGATATGTGTAG-3'	
CsPUB37-YFP-F	5'-ctgagtttttctgattaacagATGGATTCTGATTTTCCTCCTC-3'	1284 bp
CsPUB37-YFP-R	5'-gcccttgctcaccatggatccTTCATCATTCACAAACTTCAAATAATTC-3'	
CsPUB43-YFP-F	5'-ctgagtttttctgattaacagATGCAGTCTGAAGTTGAATCTC-3'	1507 bp
CsPUB43-YFP-R	5'-gcccttgctcaccatggatccGGAAGACGAAAATGTCACTCTTG-3'	
