# Supplementary material for: Genome-Wide Identification, Characterization, and Expression Analysis of the U-Box Gene Family in Cucumber (Cucumis sativus)
Source: Plants (Basel). 2025 Jun 12;14(12):1801. doi: 10.3390/plants14121801 (PMC12196615; doi:10.3390/plants14121801)
Supplement: Supplementary file 1 [file plants-14-01801-s001.zip › supplement-Figure.pdf]

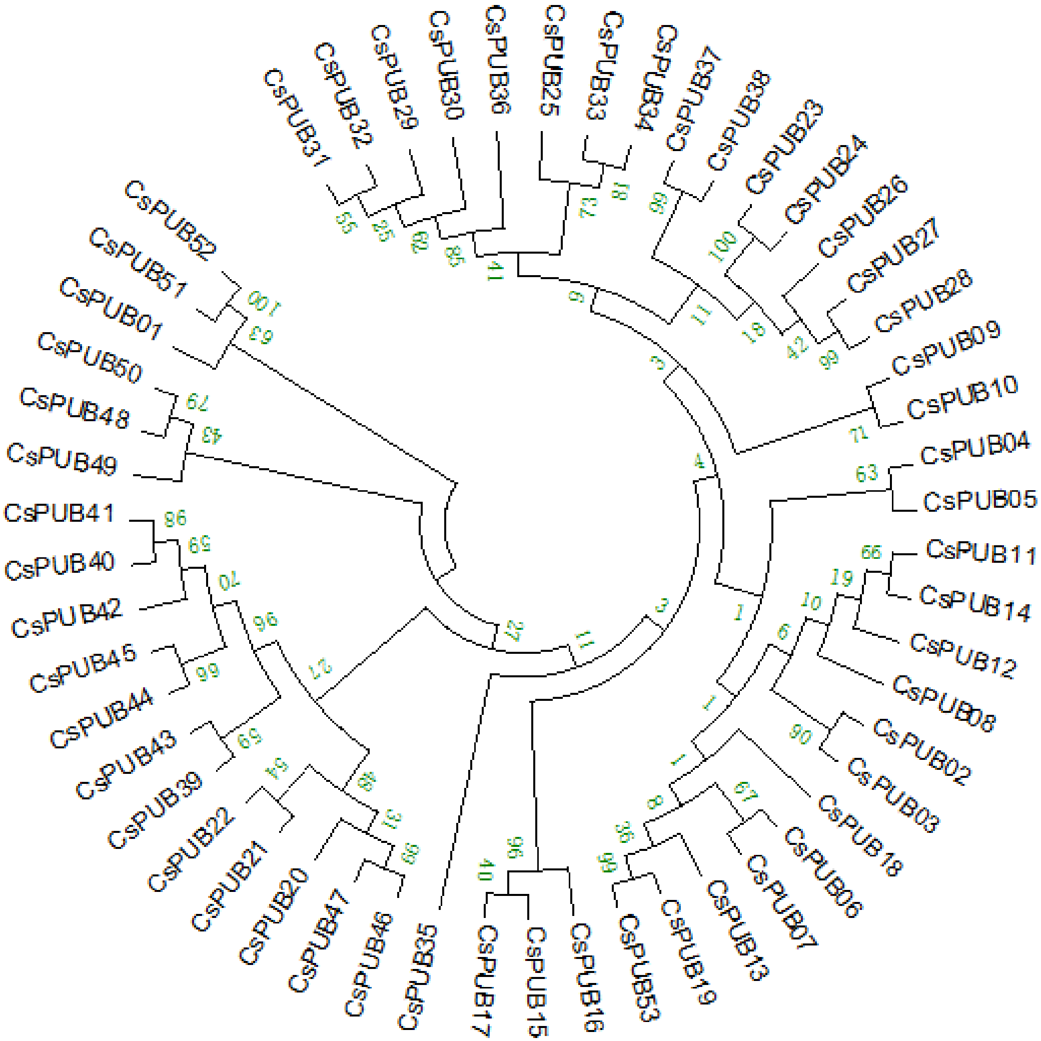

Supplement Figure S1. Evolutionary analysis of the U-box in CsPUBs.
The sequences in the figure are based solely on protein sequences containing the U-box portion.

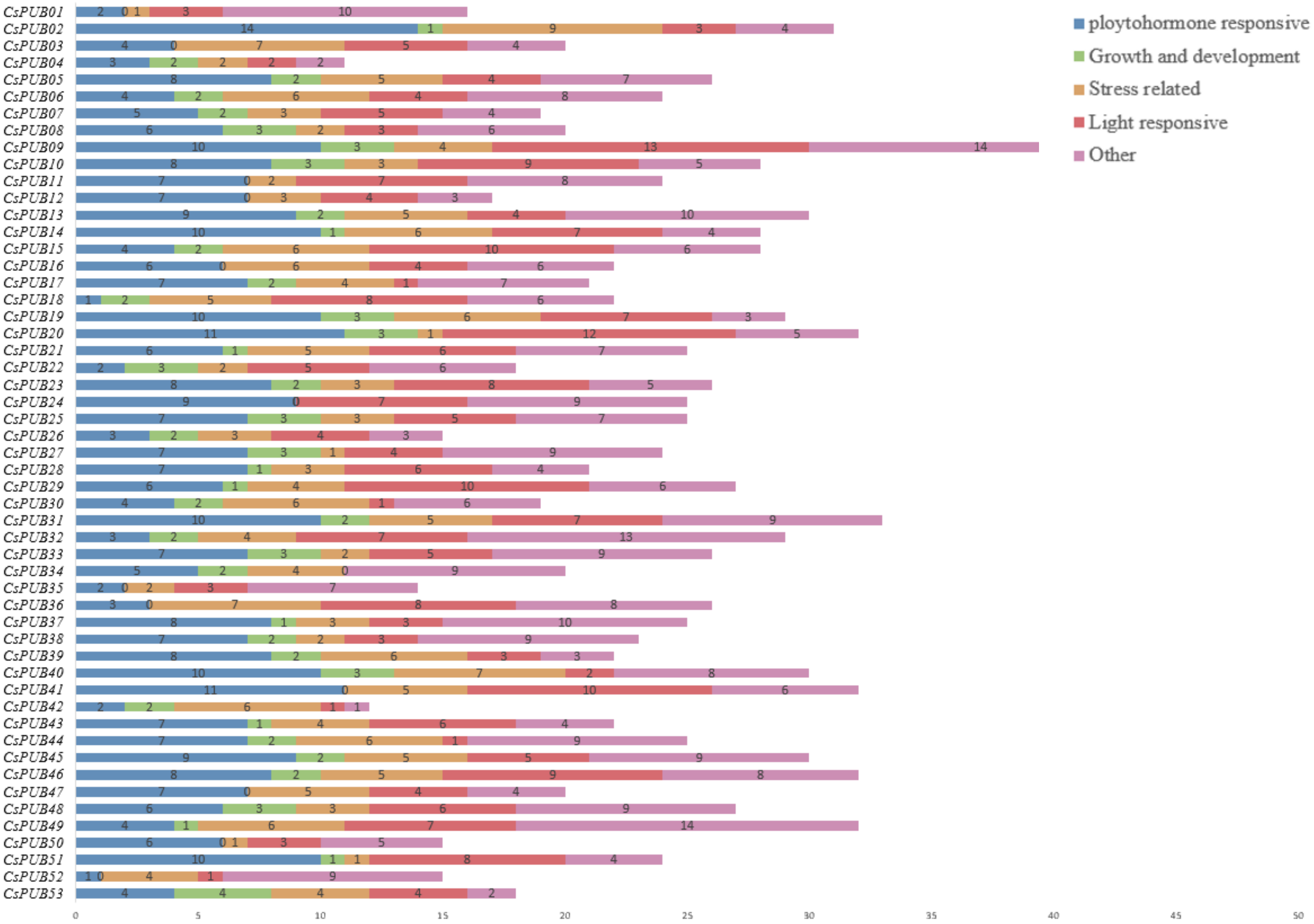

Supplement Figure S2. Statistical analysis of the number of *cis*-acting elements of *CsPUBs.*

Statistics on the number of cis-acting elements in *CsPUBs*, where blue represents plant hormone responsiveness elements, green represents growth and development-related elements, orange represents stress responsiveness-related elements, red represents light responsiveness elements, and purple represents elements with other functions.
